# Conservative Treatment of Compound Comminuted Fracture of the Femur, with Cases

**Published:** 1865-01

**Authors:** G. M. B. Maughs

**Affiliations:** Surgeon P. A. C. S.


ART. IV.—*Conservative Treatment of Compound Comminuted Fracture of the Femur, with Cases.* By G. M. B MAUGHS, Surgeon P. A. C. S.

*Case* 1.—C. S. Sheffield, private 15th Mississippi cavalry, aged 17, of feeble constitution, power of reaction very low, was wounded at Tishimingo Creek, June 10th, 1864, transported some thirty or forty miles in an ambulance to railroad, and thence in cars some 130 miles, to Lauderdale Hospital, where he was admitted June 15th, 1864; compound comminuted fracture of femur at juncture of middle and upper third—comminution very severe. Treated by Smith's anterior splint; but little effort at reparation; suppuration excessive. Died August 1st, 1864, of hectic and exhaustion.

*Case* 2.—M. B. James, private, company "H," 1st Kentucky cavalry, aged 19, wounded at Tishimingo Creek June 10th, transportation as case 1st, admitted in hospital June 19th, 1864; compound comminuted fracture of femur at upper third, the ball in its oblique course passed through the bone as high up as the trochanter major. Treated by position; attempted union by free provisional callus; doing well until the middle of August, when he was attacked with continued fever, of which he died September 9th, 1864.

*Case* 3.—W. H. Ray, private, Newsom's cavalry, company "H," aged 24, wounded at Tishimingo Creek June 10th, 1864, admitted into hospital June 19th, 1864; compound comminuted fracture of femur at middle third. Treated by Smith's anterior splint; cure perfect; no shortening or deformity.

*Case* 4.—E. H. Powell, private, Newsom's cavalry, company "H," aged 49, wounded at Tishimingo Creek June 10th, 1864, admitted June 19th, 1864; compound comminuted fracture of femur at middle third. Treated by Smith's anterior splint; recovered with three inches shortening.

*Case* 5.—W. B. Davis, private, 6th Mississippi cavalry, company "F," wounded at Harrisburg July 14th, admitted at Lauderdale Hospital July 18th; compound comminuted fracture of femur at middle third. Treated by Smith's anterior splint; union perfect; shortened one inch; no deformity otherwise.

*Case* 6.—D. C. Crouch, private, 20th Tennessee, company "F," aged 19, wounded at Harrisburg July 14th, 1864, admitted July 18th, 1864; compound comminuted fracture of femur at middle third; extensive comminution. Treated by Smith's anterior splint; furloughed October 12th, 1864; perfect union; shortened three inches.

*Case* 7.—J. Posten, private, 1st Kentucky cavalry, company "C," aged 18, wounded at Harrisburg July 14th, 1864, admitted July 18th, 1864; ball passed through both thighs; compound comminuted fracture of left femur at upper third at trochanter. Treated by Smith's anterior splint; furloughed September 26th; cure perfect; shortening scarcely perceptible.

*Case* 8.—J. H. Shelley, private, 3d Kentucky cavalry, company "L," aged 20, wounded near Tishimingo Creek June 9th, 1864, admitted June 14th; compound comminuted fracture of femur at the juncture of middle and upper third. Treated by position. The cure in this case has been greatly retarded, and the limb rendered useless by the meddlesome surgery to which he was subjected, in incising the soft parts and removing the broken fragments of the femur. Union complete; will recover with great shortening.

*Case* 9.—J. W. Martin, private, 7th Kentucky cavalry, company "K," aged 19, wounded at Tishimingo Creek June 10th, admitted June 14; compound comminuted fracture of femur at the junction of middle and upper third; extensive injury to soft parts; died June 16th, 1864.

*Case* 10.—J. L. Lawrence, private, 8th Kentucky, company "C," aged 25, wounded at Tishimingo Creek June 10th, 1864, admitted June 14th; compound comminuted fracture of femur at upper third; died June 16th, 1864.

*Case* 11.—N. L. McNight, private, 14th Confederate cavalry, wounded at Harrisburg July 15th, 1864; compound comminuted fracture of femur at upper third. Treated by long, straight splints; died September 9th, 1864.

*Case* 12.—N. L. McGoodwin, sergeant-major 3d Kentucky cavalry; compound comminuted fracture of femur at junction of middle and upper third; extensive comminution. Treated by position; union complete; shortened two and a half inches; wounded at Harrisburg July 14th, 1864; furloughed September 16, 1864.

*Case* 13.—J. T. Tindell, 18th Mississippi cavalry, company "K," aged 19, wounded July 15, 1864; compound comminuted fracture of femur at upper third, the ball passing up to the joint; extensive comminution, with great injury to soft parts; died September 18, 1864.

*Case* 14.—A. Latten, Morgan's command, wounded at Harrisburg July 15th, 1864; compound comminuted fracture of femur at upper third, near hip joint; injury to bone and soft parts very severe; wound sloughed extensively.—Treated by long splint; died of pyæmia Sept. 1st, 1864.

*Case* 15.—J. Malone, Morgan's command, aged 21, wounded at Harrisburg July 15, 1864; compound comminuted fracture of femur at middle third Treated by long splint; partial union of bone; wound doing well, when he was taken with pyæmia, and died September 17th, 1864.

*Case* 16.—V. T. Bynum, 3d Kentucky cavalry, aged 23, wounded at Harrisburg July 15th, 1864; compound comminuted fracture of femur at lower third. Treated by position; union complete; shortening two inches; gone home.

*Case* 17.—J. H. Stevens, 3d Kentucky cavalry, company "D," aged 20, wounded at Harrisburg, July 14th, 1864; compound comminuted fracture of femur, upper-third; treated by position; had pthisis pulmonalis; died August 3d, 1864.

*Case* 18.—H. Jenkins, private, company "H," Mississippi Partizan Rangers, wounded at Harrisburg, July 14th, 1864, aged 23; compound comminuted fracture of femur, middle-third; treated by position; cure perfect; shortening scarcely perceptible.

*Case* 19.—W. T. Ivy, private, 19th Mississippi cavalry, aged 40, wounded at Harrisburg, July 14th, 1864; compound comminuted fracture of femur, upper-third; ligation of arteria profunda; treated by position; union complete; shortened one inch.

*Case* 20.—W. W. Shophire, 38th Mississippi, company "D," aged 28, wounded at Harrisburg, July 14th, 1864; compound comminuted fracture of femur, junction of middle and upper-third; very great comminution; attacked with erysipelas; recovered; union complete; shortening one inch; furloughed August 12th, 1864.

*Case* 21.—Yankee Lieutenant, wounded at Harrisburg, July 14th, 1864; compound comminuted fracture of femur, upper third; during treatment femoral artery sloughed and was ligated complete recovery; has been exchanged.

2

*Case* 22.—A. Bay, attached to the army, was admitted for wound of femur, middle-third, compound comminuted; treated by position; union complete; but little deformity; gone home.

Here we have twenty-two cases of compound comminuted fracture of the femur, treated without amputation; not selected to make an argument, but consecutive cases, and include, with a single exception, all the cases so wounded and so treated at these hospitals, during the time included within this report. And we have not extended the report through a greater time, or hunted up isolated cases because others might have been wounded at the same time, of whom no record was preserved, thereby rendering the statistics incomplete and worthless.

To the statistician, these twenty-two cases are worth more than a thousand would be, gathered up from different sections, where in each section a few cases had been omitted, and the result in others was not known. Such statistics are like native offerings in heathen temples, they tell but a partial story.

The excepted case is that of a private, wounded at Harrisburg by a grapeshot through both thighs near the hip-joint, with great destruction of the soft parts, tearing out the testicles, and bruising the perineum. It was not expected that this unfortunate man could survive even a few days. His testicles were removed, the wounds dressed, and his mangled limbs placed in the most comfortable position; stimulants and nourishing diet were administered. He lived for six weeks, during which time one of the wounds healed up, the bone united, and considerable progress had been made in the other limb, when he sank, worn out with the extensive suppuration.

This case is mentioned and fairly stated to show that it could not have been included with those given in this report, as the object of this paper is not to prove that wounds of the thigh may not be of such a nature as to require amputation. Would, however, the most reckless advocate of the knife have used it in this case?

Our object is to prove that in all compound comminuted fractures of the femur, with the artery intact and no very great destruction of soft parts, conservative surgery not only gives the patient the advantages of a natural over an artificial limb, but also gives him a better chance for his life than amputation would. And all of this we think this paper establishes.

Of these twenty-two cases nearly all were subjected to circumstances most unfavorable to recovery. The patients from the Tishimingo battle, treated by Assistant-Surgeon S. Kennedy, were hauled over rough roads for thirty or forty miles, and then transported some hundred and thirty miles by railroad, being constantly disturbed and their wounds fretted for eight or nine days before their arrival at Lauderdale hospital. Several of the patients from the battle of Harrisburg were subjected to nearly the same transportation and frequent change of place; the delay, however, in their arrival at the hospital was not so great; some of the others were necessarily removed three or four times during their treatment. The surgeon in charge, Dr. Hoyle, thinks the death of more than one of these clearly attributable to this untoward circumstance. Cases 9 and 10 died soon after their admission, never having re-acted from the shock. Case 17 had pthisis pulmonalis, of which he most probably died; admitting, however, that he did not really die of this, it can not be denied that it rendered his recovery from any serious injury most improbable. Case 2 was progressing favorably when attacked with continued fever, at the time prevailing in the hospital, of which he died. While, therefore, there would be much justification for excluding cases 2, 9, 10 and 17 from the report, as their deaths were only indirectly the result of their wounds, yet we will give the advocates of amputation the benefit of them.

We have then recovered, cases 3, 4, 5, 6, 7, 8, 12, 16, 18, 19, 20, 21, 22—total, 13, or $59\frac{1}{11}$ per cent. Died, cases 1, 2, 9, 10, 11, 13, 14, 15, 17—total, 9, or $40\frac{10}{11}$ per centum.

Now, let us see what would have been the result had amputation been performed in all those cases. In cases 2, 7, 13, amputation must have been at hip-joint; disarticulation, all of whom would have died. That a case does now and then survive this formidable operation does not affect the rule that of ten or twenty so operated upon all will die. Dr. Macleod never saw a successful case in military surgery. We have heard of but one—that of a private wounded by a shell at Fort Pemberton, and operated upon by Surgeon W. M. Compton, and afterwards attended by Surgeon Green in hospital at Yazoo city.

In cases 1, 8, 9, 10, 11, 12, 13, 14, 17, 19, 20, 21—total, 12—amputation must have been high up in upper third, through or near the trochanters; of these, nine, most probably ten, would have died. In the other seven cases, amputation could not have been lower than the middle third; of these, four would have died, giving for operative surgery, deaths 16 or $72\frac{8}{11}$ per cent., recoveries 6 or $27\frac{3}{11}$ per cent., or more than 100 per cent. in favor of conservative, under the most untoward circumstances, over operative surgery, under circumstances the most favorable.

And this proportion, we doubt not, but for the meddlesome interference of the surgeons, would hold good throughout the Confederacy, or even be greatly increased, as it would scarcely happen that a concatenation of circumstances so unpromising would again be met with. It will be observed that in all of these cases, with a single exception, and that greatly to the detriment of the patient, the treatment was eminently conservative. *No* EXCISIONS of the *femur, no* INCISIONS *of soft parts for the removal* of loose pieces *of bone*, no formidable display of machinery to keep the limb *in* place and the patient *from* sleep.

The wounds were carefully examined, and all foreign bodies, including spiculæ of bone immediately in the track of the ball, removed. The limb was placed in Smith's anterior splint, or simply a straight splint, or what was preferred, placed in position and retained there by soft cushions or pillows, and all unnecessary probing or handling carefully voided.

By even late authorities, the experience of the Crimean campaign and Dr. Macleod's observations have been quoted to prove that ordinary fractures from rifle balls, above the knee, (of the femur,) demand amputation. These give only eight per cent. recoveries for conservative surgery under the most favorable circumstances, with selected cases, and only thirty-two per cent. for amputation. This is indeed an alarming fatality, and as frightfully dangerous, as it shows amputations of the thigh to be two to one against recovery, as it is four times more successful than conservative treatment, would of course establish the rule, to amputate. But against such a rule our paper is an unanswerable demurrer; and as it reverses the *result*, and proves that by saving the limb we save twice as many lives as could be saved by amputation, by the same parity of reasoning it must also reverse the *rule*, and establish this. *As a general rule, ordinary fractures above the knee, from rifle balls, should never cause primary amputation.*

